# Pregnancy-Associated Heart Failure: A Comparison of Clinical Presentation and Outcome between Hypertensive Heart Failure of Pregnancy and Idiopathic Peripartum Cardiomyopathy

**DOI:** 10.1371/journal.pone.0133466

**Published:** 2015-08-07

**Authors:** Ntobeko B. A. Ntusi, Motasim Badri, Freedom Gumedze, Karen Sliwa, Bongani M. Mayosi

**Affiliations:** 1 The Cardiac Clinic, Department of Medicine, Groote Schuur Hospital and University of Cape Town, Cape Town, South Africa; 2 Department of Statistical Sciences, University of Cape Town, Cape Town, South Africa; 3 Hatter Institute for Cardiovascular Research in Africa, Department of Medicine, Groote Schuur Hospital and University of Cape Town, Cape Town, South Africa; Virginia Commonwealth University, UNITED STATES

## Abstract

**Aims:**

There is controversy regarding the inclusion of patients with hypertension among cases of peripartum cardiomyopathy (PPCM), as the practice has contributed significantly to the discrepancy in reported characteristics of PPCM. We sought to determine whether hypertensive heart failure of pregnancy (HHFP) (i.e., peripartum cardiac failure associated with any form of hypertension) and PPCM have similar or different clinical features and outcome.

**Methods and Results:**

We compared the time of onset of symptoms, clinical profile (including electrocardiographic [ECG] and echocardiographic features) and outcome of patients with HHFP (n = 53; age 29.6 ± 6.6 years) and PPCM (n = 30; age 31.5 ± 7.5 years). The onset of symptoms was postpartum in all PPCM patients, whereas it was antepartum in 85% of HHFP cases (p<0.001). PPCM was more significantly associated with the following features than HHFP (p<0.05): twin pregnancy, smoking, cardiomegaly with lower left ventricular ejection fraction on echocardiography, and longer QRS duration, QRS abnormalities, left atrial hypertrophy, left bundle branch block, T wave inversion and atrial fibrillation on ECG. By contrast, HHFP patients were significantly more likely (p<0.05) to have a family history of hypertension, hypertension and pre-eclampsia in a previous pregnancy, tachycardia at presentation on ECG, and left ventricular hypertrophy on echocardiography. Chronic heart failure, intra-cardiac thrombus and pulmonary hypertension were found significantly more commonly in PPCM than in HHFP (p<0.05). There were 5 deaths in the PPCM group compared to none among HHFP cases (p = 0.005) during follow-up.

**Conclusion:**

There are significant differences in the time of onset of heart failure, clinical, ECG and echocardiographic features, and outcome of HHFP compared to PPCM, indicating that the presence of hypertension in pregnancy-associated heart failure may not fit the case definition of idiopathic PPCM.

## Introduction

Peripartum cardiomyopathy (PPCM) is defined as a myocardial disorder of unknown cause, characterized by marked impairment of left ventricular (LV) systolic function, with development of heart failure towards the end of pregnancy and in the months following delivery, in women without pre-existing heart disease, and in the absence of any other identifiable cause of peripartum heart failure [1,2]. Although the aetiology of PPCM is poorly understood [3,4], many authorities consider pregnancy-induced hypertension (PIH) to be a risk factor for PPCM [4–8]. Furthermore, it has been postulated that hypertensive heart failure of pregnancy (HHFP), defined as the occurrence of peripartum heart failure in association with any form of hypertension, and PPCM may represent a spectrum of the same disease which has a common pathophysiological pathway [9].

Our group has proposed a clear case definition of PPCM, noting that PPCM should be a diagnosis of exclusion that precludes all other known causes of peripartum heart failure, including hypertension [3,10]. Since the seminal description of PPCM by Demakis in 1971, where preeclampsia was found in 22% of the 27 patients studied [5], it has been unclear what the role of PIH in PPCM is. Indeed, many authors have described great variability in the prevalence of PIH in PPCM, with preeclamptic patients accounting from 15 to 89% of PPCM patients reported in the different studies [5–8, 11–16]. It is has been suggested that the inclusion of patients with varying degrees of gestational hypertension, in the index as well as previous pregnancies, has contributed significantly to the discrepancy in reported characteristics of PPCM [2,3,17].

We have performed a study of the clinical characteristics and outcome of a consecutive series of patients with a new diagnosis of pregnancy-associated heart failure occurring in the last trimester of pregnancy or puerperium in Cape Town. The study was conducted during a period of great social and epidemiological transition in South Africa, shortly after the introduction of democracy. We investigated whether there were significant differences in time of onset of symptoms, clinical profile, and outcome between HHFP cases compared to patients with unexplained PPCM, in order to assess whether they can be considered to be a spectrum of the same disease, or whether they should be classified differently.

## Methods

### Study design

This is a prospective, longitudinal hospital-based case-comparison study of HHFP to PPCM, in patients presenting with heart failure between the last month of pregnancy and the fifth postpartum month, who had been referred to the Cardiac Clinic at Groote Schuur Hospital in Cape Town, South Africa.

We invited clinicians to refer patients with a new diagnosis of heart failure occurring in the last trimester or puerperium for enrollment into a study of risk factors of PPCM. The exclusion criteria were a known cardiac lesion; in particular, valvular heart disease, previous anthracycline exposure, ischaemic heart disease, a congenital heart lesion, and metabolic and systemic disorders with cardiovascular sequelae including diabetes mellitus and thyroid disease. Patients with any form of hypertension in pregnancy (including PIH) were included in the study; the latter patients were classified as HHFP. The study participants were evaluated by one physician (BMM) consecutively between February 1, 1996 and December 31, 2009 in a specialist cardiomyopathy clinic. The median follow-up for PPCM was 3.5 years and 6 years for HHFP.

### Definition of HHFP and PPCM

The diagnosis of HHFP was based on the presence of clinical heart failure associated with any form of hypertension (i.e., chronic hypertension, gestational proteinuric hypertension, preeclampsia, eclampsia and postpartum hypertension), occurring in women between the last month of pregnancy and the first 5 months of the postpartum, in the absence of pre-existing heart disease, and any other identifiable cause of peripartum heart failure besides hypertension. Even though most of this group of study subjects had evidence of depressed systolic function (LVEF less than 45%) shortly after delivery, on echocardiography, there was no restriction on subject selection based on echocardiographic parameters.

The standard definition of PPCM was applied; the patients with PPCM needed to fulfill echocardiographic criteria of a left ventricular ejection fraction (LVEF) below 45%, left ventricular fractional shortening below 28%, and a left ventricular end diastolic dimension greater than 5.5cm or greater than 2.7cm/m^2^ [1]. Patients with any form of hypertension, preeclampsia or eclampsia were excluded from the PPCM group.

### Data collection

There were 36 patients with PPCM that were enrolled in the study and 6 of these were subsequently lost to follow-up, and were not included in the analysis. All patients with a diagnosis of HHFP were included in the analysis. All patients had comprehensive clinical assessment, complemented by chest radiography, electrocardiography (ECG), two-dimensional and Doppler colour-flow echocardiography, and cardiac catheterisation, when appropriate. Blood was taken for full blood count, serum creatinine, urea and electrolytes. The primary imaging modality used to confirm the diagnosis was transthoracic two-dimensional and Doppler echocardiography. As the analysis period for the study is over 14 years, the echocardiographic assessments were performed by different cardiologists and sonographers at different time points in the follow-up, and some of these echocardiographic studies were incomplete. In the end, we were able to establish the vital status of 30 PPCM and 53 HHFP patients at the end of the follow-up period.

### Statistical analysis

Results of quantitative measurements are given as means ± SD. Categorical traits are represented as number and percentage. Pearson’s chi-square or Fisher’s exact test were used to compare the relative frequency of characteristics between the two groups of patients. All P values are two-sided; and P values < 0.05 are considered to be statistically significant. Survival analysis was performed using Kaplan-Meier plots.

### Ethics statement

This study was approved by the University of Cape Town Faculty of Health Sciences Human Research Ethics Committee. All patients gave informed consent for participation in the study, and the study complies with the Declaration of Helsinki.

## Results

### Baseline characteristics

**Fig 1:**
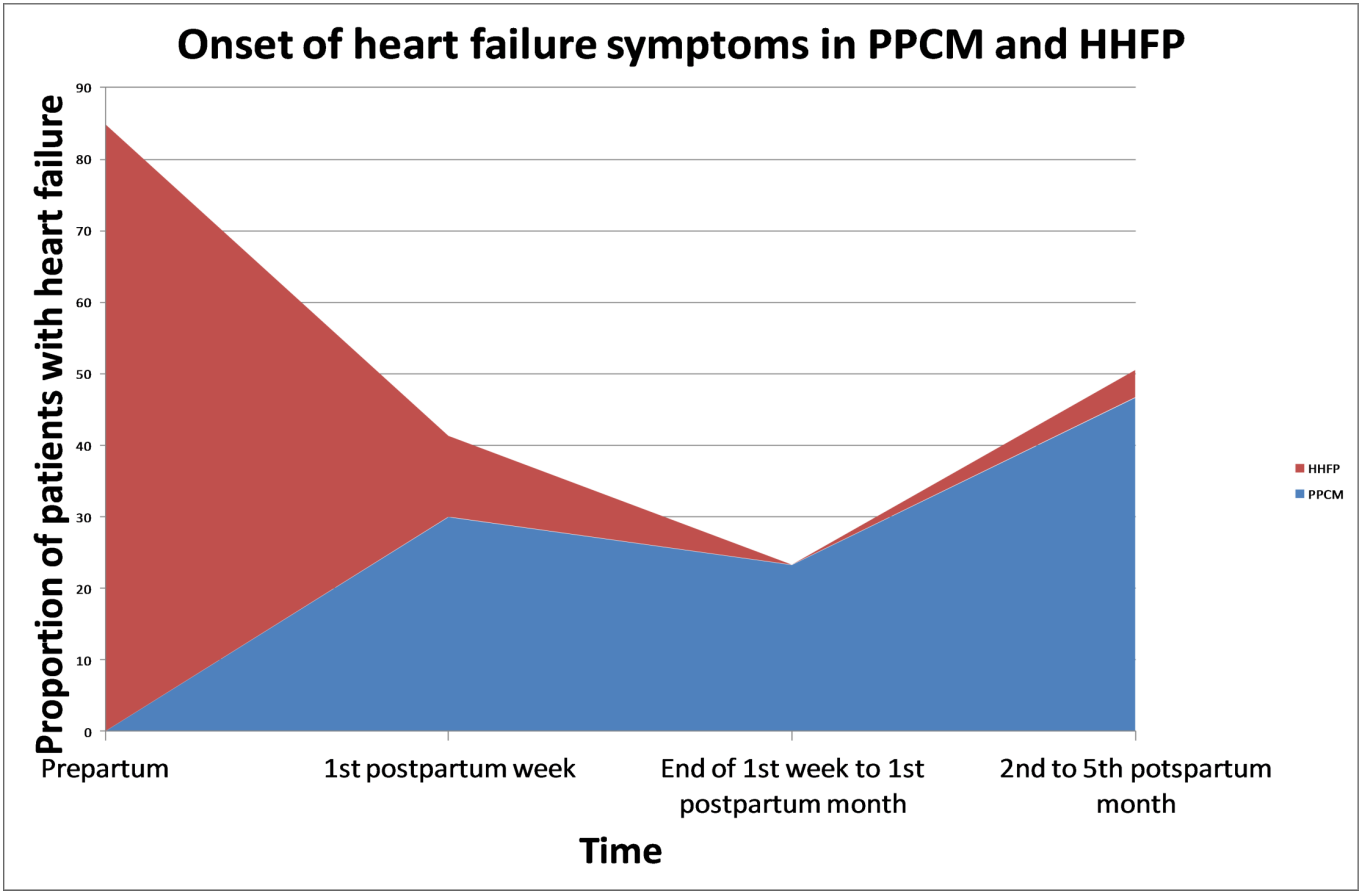
Onset of heart failure symptoms in hypertensive heart failure of pregnancy and peripartum cardiomyopathy.

A total of 83 female patients were included in this study (Table 1). PPCM patients had an average age of 31.5 ± 7.5 years and HHFP patients had a similar mean age of 29.6 ± 6.6 years (p = 0.223). Nine (30%) of PPCM patients developed heart failure in the first week after delivery, 7 (23.3%) after the first week but before the end of the first postpartum month, and 14 (46.7%) after the first month, but before the end of fifth postpartum month. In contrast, the HHFP patients presented earlier, with 45 (84.9%) developing heart failure in the last month of pregnancy, 6 (11.3%) in the first postpartum week, and 2 (3.8%) between the first and fifth postpartum months (p<0.001) (Fig 1). Twin pregnancy occurred more commonly in PPCM patients (p = 0.044), as did smoking (p = 0.024). A family history of hypertension (p<0.001) and a history of hypertension in a previous pregnancy (p<0.001) were found more commonly in HHFP patients.

**Table 1:** Clinical characteristics of patients with peripartum cardiomyopathy (PPCM) and hypertensive heart failure of pregnancy (HHFP) at the initial presentation with heart failure.

Clinical characteristics	PPCM (N = 30)	HHFP (N = 53*)	P-value
Ethnicity:			0,820
Black/African	18 (60.0)	30 (56.6)	
Coloured/Mixed ancestry	12 (40.0)	23 (43.4)	
Age at diagnosis	31.5 ± 7.5	29.6 ± 6.6	0.223
Onset of symptoms in relation to pregnancy:			<0.001
Last trimester	0 (0)	45 (84.9)	
Within 1^st^ week after delivery	9 (30.0)	6 (11.3)	
> 1^st^ week,<1^st^ month after delivery	7 (23.3)	0 (0)	
> 1^st^ month,<5^th^ month after delivery	14 (46.7)	2 (3.8)	
Twin pregnancy	3 (10.0)	0 (0)	0.04
Family history of hypertension	3 (10.0)	35 (66.0)	<0.001
History of hypertension in previous pregnancy	0 (0)	17 (32.1)	<0.001
NYHA FC at presentation:			0,163
Class I and II	10 (33.3)	9 (16.9)	
Class III and IV	20 (66.7)	44 (83.1)	
Pedal oedema	24 (80.0)	19 (35.8)	<0.001
Parity	2.4 ± 0.7	2.2 ± 0.6	0.591
Gravidity	2.4 ± 0.7	2.2 ± 0.6	0.595
Smoking:			0,24
Never smoker	21 (70.0)	33 (62.3)	
Former smoker	2 (6.7)	15 (28.3)	
Current smoker	7 (23.3)	5 (9.4)	
Alcohol:			0,244
Never drinker	23 (76.7)	45 (89.4)	
Former drinker/moderate use	4 (13.3)	7 (13.2)	
Excessive intake	3 (10.0)	1 (1.9)	
HIV seropositive	3 (10.0)	6 (11.3)	0.582
Delay from symptom onset to clinical assessment (months)	2.7 ± 1.4	1.1 ± 0.3	<0.001
Systolic blood pressure	105.9 ± 16.2	162.3 ± 28.4	0.003
Diastolic blood pressure	63.5 ± 9.6	105.0 ± 12.1	<0.001
Basal rales	16 (53.3)	41 (77.4)	0.007
Murmur:			0,006
No murmur	10 (33.3)	35 (66.0)	
MR	13 (43.3)	14 (24.6)	
MR + TR	7 (23.3)	3 (5.7)	
ESM	0 (0)	1 (1.9)	

(*48 [90.6%] of the 53 HHFP patients had a diagnosis of preeclampsia; NYHA FC = New York Heart Association functional class; MR, mitral regurgitation; TR, tricuspid regurgitation; ESM, ejection systolic murmur).

### Clinical characteristics at presentation

Three (10%) of the PPCM patients and 6 (11.3%) of the HHFP patients were HIV-infected (p = 0.582). The systolic and diastolic blood pressures measurements were 105.9 ± 16.2 mmHg and 63.5 ± 9.6 mmHg in PPCM patients and 162.3 ± 28.4 mmHg and 105.0 ± 12.1 mmHg in HHFP patients (p = 0.003 and p<0.001, respectively). Basal rales were detected in 16 (53.3%) of PPCM patients and 41 (77.4%) of HHFP patients (p = 0.007). Peripheral oedema was present in 80% of PPCM patients compared to 35.8% of HHFP patients (p<0.001).

### Radiographic, electrocardiographic, and echocardiographic features

A cardiothoracic ratio (CTR) greater than 50% was found more commonly in PPCM compared to HHFP patients (p<0.001) (Table 2). Radiographic evidence of pulmonary oedema was noted more frequently in HHFP patients, in keeping with the clinical detection of rales (p = 0.010). Higher heart rate was found in HHFP patients (p = 0.014). On electrocardiography (ECG), atrial fibrillation (p = 0.028), QRS abnormalities (p = 0.001), relatively longer QRS duration (p<0.001), left atrial hypertrophy (p = 0.030), and left bundle brunch block (p = 0.002), and T wave inversion (p<0.001) were detected more commonly in PPCM patients than in HHFP patients. As expected, left ventricular hypertrophy (LVH) (p = 0.021) was seen more frequently in HHFP patients. On echocardiography, patients with HHFP had greater ventricular septal thickness in diastole (p = 0.013) as well as greater LV posterior free wall thickness in systole (p = 0.002). PPCM patients had larger LV dimensions both in systole and diastole (p<0.001 and p<0.001, respectively), as well as lower LVEF and LV fractional shortening (p<0.001 and p<0.001, respectively) compared to HHFP patients.

**Table 2:** Radiologic, electrocardiographic, and echocardiographic findings at initial presentation with heart failure.

Radiologic, electrocardiographic, echocardiographic and laboratory findings	PPCM (N = 30)	HHFP (N = 53)	P-value
Radiographic cardiothoracic ratio>50%	29 (96.7)	22 (41.5)	<0.001
Radiographic pulmonary oedema	16 (53.3)	45 (89.4)	0.010
ECG heart rate	86.3±17.8	106.9±27.4	0.014
ECG QRS abnormalities	8 (27.6)	1 (1.9)	0.001
ECG voltage abnormality	8 (27.6)	31 (58.5)	0.816
ECG left anterior hemiblock	9 (30.0)	5 (9.4)	0.03
ECG Q wave	5 (16.7)	5 (9.4)	0.484
ECG left bundle branch block	6 (20.0)	0 (0)	0.002
ECG Left ventricular hypertrophy	8 (26.7)	29 (54.7)	0.021
ECG T wave inversion	22 (73.3)	13 (24.5)	<0.001
Atrial fibrillation	3 (10.0)	0 (0)	0.028
QRS duration	109.5±12.17	89.8±10.26	<0.001
Echo interventricular septum (diastole)	0.9 ± 0.2	1.2 ± 0.2	0.013
Echo LV posterior wall (systole)	1.2 ± 0.3	1.4 ± 0.3	0.002
Echo LV end-systolic diameter (diastole)	6.8 ± 0.7	3.5 ± 0.6	<0.001
Echo LV end-diastolic dimension (diastole)	7.4 ± 1.1	5.1 ± 0.9	<0.001
Echo LV ejection fraction	23.8 ± 8.3	49.9 ± 18.7	<0.001
Echo LV fractional shortening	11.6 ± 4.1	26.2 ± 3.2	<0.001

### Medical management at follow up

**Fig 2:**
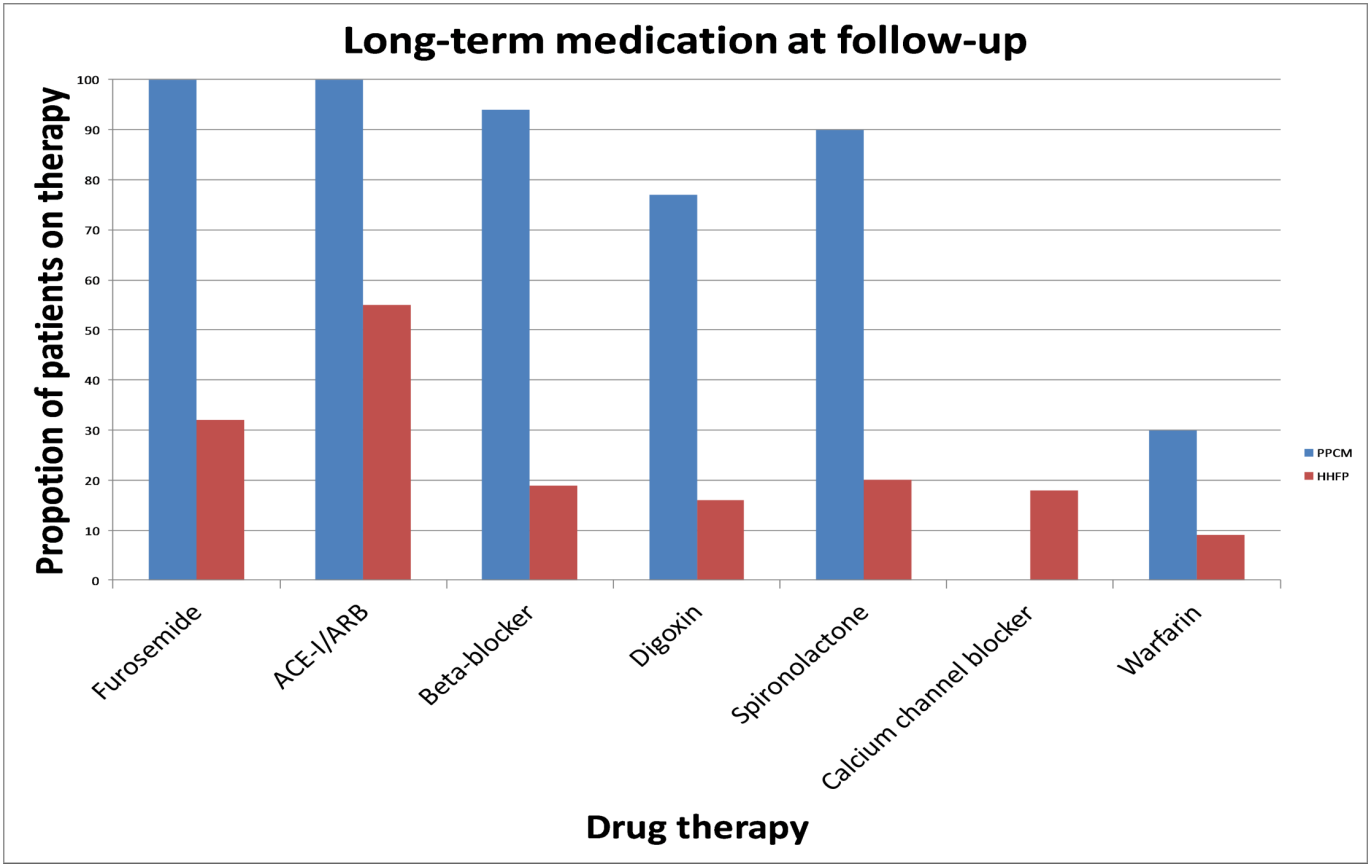
Use of medication at follow-up.

With regards to heart failure therapy, significantly more PPCM patients were receiving furosemide (p<0.001), angiotensin converting enzyme-inhibitor (ACE-I)/angiotensin receptor blocker (ARB) (p<0.001), β-blockers (p<0.001), spironolactone (p<0.001) and digoxin (p<0.001) compared to HHFP patients (Fig 2). However, more HHFP patients were on calcium channel blockers (CCB) for treatment of hypertension than PPCM (p = 0.014). Warfarin was prescribed more commonly for consequent atrial fibrillation, and LV thrombus for PPCM patients (p = 0.030).

### Survival analysis

**Fig 3:**
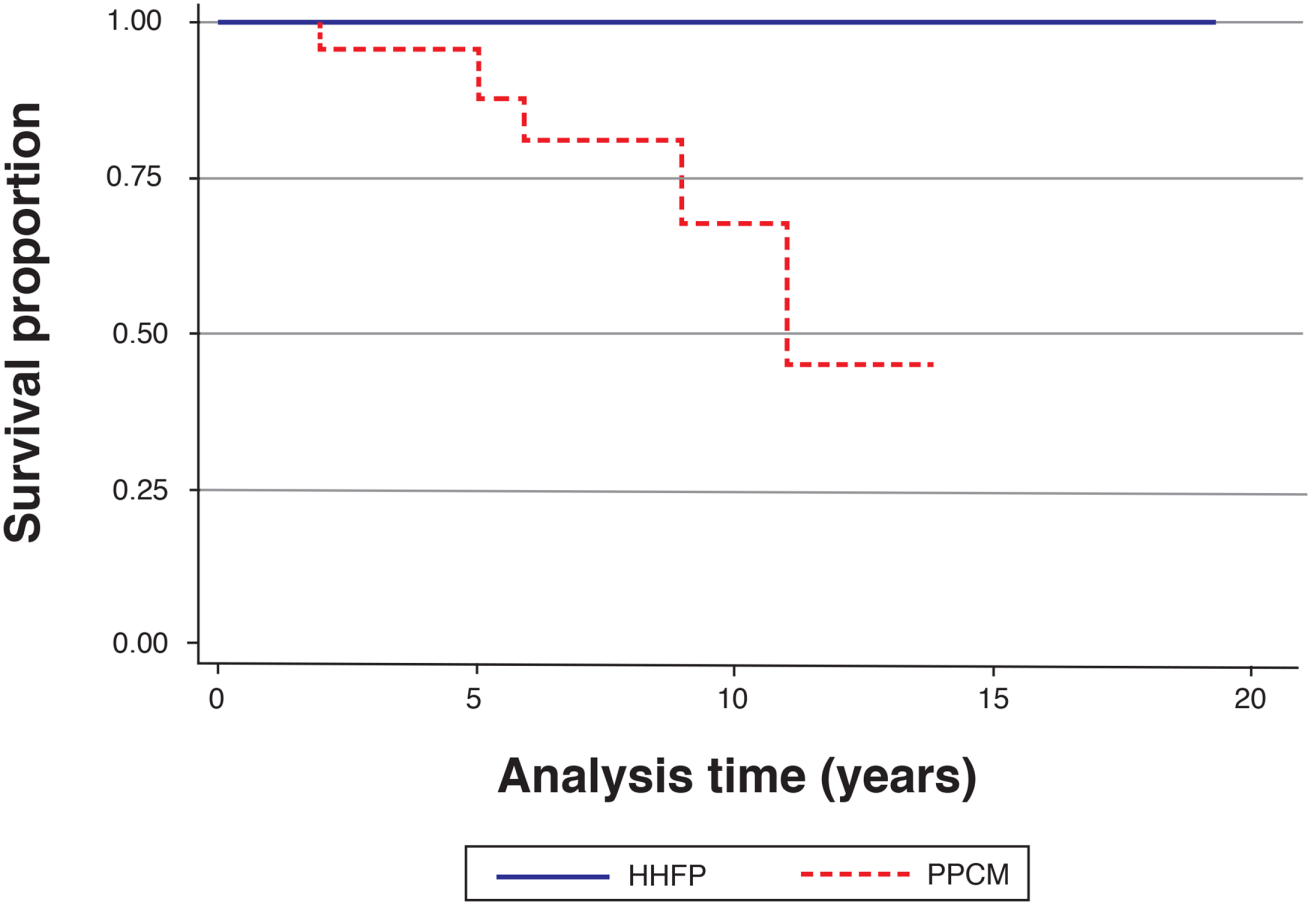
Kaplan-Meier survival plot for hypertensive heart failure of pregnancy and peripartum cardiomyopathy patients over 14 years.

There were 5 deaths in PPCM patients during the 14 years of follow-up, while there were no deaths in the HHFP group (p = 0.005). A Kaplan-Meier analysis of survival stratified according to PPCM versus HHFP at last follow-up visit (p<0.001) is shown in Fig 3. Table 3 shows that chronic heart failure was more common in PPCM patients at the last follow-up visit (p<0.001). Similarly, intra-cardiac thrombus (p = 0.014) and development of pulmonary hypertension (p = 0.022) were commoner in PPCM patients. At the most recent hospital visit, 18 (72.0%) of PPCM patients were in NYHA functional class I and II and 7 (28.0%) were in functional class III and IV compared to 52 (98.1%) of HHFP patients with class I and II symptoms and 1 (1.9%) with class III and IV symptoms (p<0.001).

**Table 3:** Survival and status at last follow-up for the PPCM and HHFP patients studied.

	PPCM (N = 30)	HHFP (N = 53)	P-value
Median duration of follow-up	3.5 years	6 years	0.02
Death	5 (16.7)	0 (0)	0.005
Chronic heart failure	24 (80.0)	8 (15.1)	<0.001
Cardiac resynchronization therapy	1 (3.3)	0 (0)	0.361
Intracardiac thrombus	5 (16.7)	0 (0)	0.014
Stroke	2 (6.7)	0 (0)	0.128
Pulmonary hypertension	4 (13.3)	1 (1.9)	0.022
Heart transplantation	1 (3.3)	0 (0)	0.361
NYHA FC at last visit:			<0.001
Class I and II	18 (72.0)	52 (98.1)	
Class III and IV	7 (28.0)	1 (1.9)	

NYHA FC, New York Heart Association Functional Class.

## Discussion

We present the results of a case comparison study of the clinical features and outcome of pregnancy-associated heart failure with or without hypertension in patients with no history of structural heart disease and show that in our centre unexplained peripartum cardiomyopathy is exclusively a postpartum disease that is associated with distinct clinical features of heart muscle disease (such as left bundle branch block and atrial fibrillation), chronic heart failure, and a fatal outcome in a proportion of cases. By contrast, HHFP is predominantly an antepartum disease that presents mainly with pulmonary oedema, left ventricular hypertrophy, and a reversible form of heart failure in the overwhelming majority of cases.

We have found that PPCM to be a postpartum condition, with 53% of PPCM patients presenting within the first month of the puerperium and the other 47% presenting within the subsequent four postpartum months, in contrast to HHFP, where 85% of patients presented with heart failure in the last month before delivery. Our findings are similar to those of another South African study which found that heart failure symptoms develop in the postpartum period in 100% of PPCM patients [18]. Summarising pooled data from 419 cases of PPCM, Lampert and Lang, demonstrated that 78% of PPCM cases developed symptoms in the first four months postpartum, while 9% had their onset of symptoms in the last antepartum month, and 13% either developed symptoms before one month antepartum or after four months postpartum [19]. In the main, however, studies comprising a greater proportion of patients with preeclampsia have documented greater frequency of onset of PPCM in the last month of pregnancy [20,21]. By comparison, studies with fewer cases of pregnancy-associated hypertension have reported a largely postpartum onset of PPCM [2,5,8,22].

A family history of hypertension was found more commonly in HHFP patients compared to PPCM patients; and in this study, a history of previous hypertension in pregnancy was present in a third of patients with HHFP and absent in those with PPCM. Genetic factors play a role in the development of pregnancy-induced hypertension (PIH), with both maternal and paternal genetic contributions being important [23,24]. Genetic predisposition to PIH/preeclampsia is suggested by the observation that primigravid women with a family history of preeclampsia (i.e., affected mother or sister) have a 2- to 5-fold increased risk of developing the disease than primigravid women without such a history [25]. Furthermore, the spouse of men who are the product of a pregnancy complicated by preeclampsia are more likely to develop preeclampsia than spouses of men without such a history [26].

Features of congestive heart failure (pedal oedema, elevated jugular venous pressure, and basal rales) were found in up to 80% of PPCM patients, but only in 35.8% of HHFP patients at presentation. In contrast, isolated pulmonary oedema was one of the main clinical and radiologic findings in patients with HHFP in this study. The association of PIH/preeclampsia with isolated pulmonary oedema is well-established [27,28]. Differences in clinical presentation between PPCM and HHFP may also be partly explained on the basis of volume overload in HHFP patients with systolic and diastolic dysfunction [29]. The pathophysiology of pulmonary oedema in preeclampsia is unclear, but is presumed to be due to a combination of microangiopathy, capillary leak and sometimes iatrogenic fluid overload [30]. PPCM, on the other hand, results from development of primary myocardial dysfunction leading to congestive heart failure [3,4], and has been shown recently, in a mouse model, to be associated with defective cathepsin-D mediated cleavage of prolactin into a 16-kDa form, which is both pro-apoptotic and anti-angiogenic [31]. PPCM may also have genetic underpinnings, and recent reports have supported the contention that PPCM may be part of familial dilated cardiomyopathy [32,33].

Multiple differences were noted on ECG in the comparison between PPCM and HHFP. First, LVH was found to occur more commonly in HHFP than in PPCM. PIH represents a model of acute pressure overload that may induce dramatic changes in left ventricular structure and function [34]. Other important ECG findings from our study included the observation that QRS abnormalities, left atrial hypertrophy, left bundle branch block (LBBB), atrial fibrillation, non-specific T wave inversion and longer QRS duration occurred more frequently in patients with PPCM compared to those with HHFP. Similar findings have been reported previously: repolarisation changes have been reported in 47.3% [35] and left bundle branch block to occur in 10% of PPCM patients [36]. Davidson and Parry found arrhythmias in 2%, and LBBB in 5% of patients with peripartum heart failure, while LVH was present in 26% and T wave changes found in 15% [37]. In a study of 97 PPCM patients from South Africa, LVH was demonstrated in 66% and ST segment and T wave abnormalities noted in 96% [38]. These ECG changes are associated with increased cardiovascular morbidity and mortality in patients with impaired LV function, and QRS duration has been shown to be strongly associated with atrial fibrillation and adverse outcome in patients with cardiomyopathy [39]. The electrocardiographic findings in our study may signify greater myocardial injury in patients with PPCM compared to those with HHFP. While the ECG of most women with PPCM is usually abnormal [2,40–43], there are no ECG changes that are sufficiently sensitive or specific for the diagnosis of PPCM [20], nor are there ECG characteristics that serve as a differentiator between PPCM and HHFP.

A higher heart rate and blood pressure were found in patients with HHFP compared to those with PPCM. The finding of increased and/or highly variable pulse rates and elevated arterial pressures in PIH/preeclampsia has been described, and is thought to partly reflect disturbed neural control of heart rate and blood pressure, as a result of impaired sympathetic and parasympathetic nervous system activity in PIH [44]. Maladaptation of the maternal cardiovascular system in PIH/preeclampsia is manifested as a lack of physiological decline in cardiovascular oscillations of heart rate and blood pressure [45].

On echocardiography, we found the interventricular septum (IVS) thickness and left ventricular posterior free wall (LVPW) thickness to be increased in HHFP compared to PPCM, likely reflecting left ventricular adaptation to increased wall stress from elevated blood pressure. Similarly, IVS and LVPW thickness were increased in PIH/preeclampsia patients compared with normal controls [46]. PIH was associated with an abnormal left ventricular (LV) geometric pattern in 62% of 106 patients studied, of which 42% had eccentric hypertrophy, 17% had concentric remodeling, and 5% had concentric hypertrophy [47]. Again, likely reflecting greater myocardial injury, the LVEF and LV fractional shortening were lower in PPCM patients and were associated with increased LV dimensions in systole and diastole. Similarly, demonstrating impaired LV function (i.e., depressed LVEF, LV cardiac output and stroke volume) and increased LV dimensions have been reported in PPCM by many authors [2,6–8]. Furthermore, the LVEF and LVEDD have been correlated with outcome in PPCM [16,48], but not in this study.

Finally, we showed that morbidity and mortality was higher in PPCM, with lack of full recovery of cardiac function (average LVEF 23.8% at diagnosis and 31.3% at last follow-up), compared to HHFP patients (average LVEF 49.9% at diagnosis and 68.2% at last follow-up). Similar findings have recently been made by Kamiya and colleagues who examined the clinical profile of Japanese PPCM patients with and without gestational hypertension and found that patients with pregnancy-associated hypertension diagnosed with PPCM had a shorter hospitalization and higher LVEF at last follow-up when compared to the PPCM patients without hypertension [48]. The mortality for PPCM of 17% over a median of 3.5 years of follow-up is similar to that seen in other countries such as Haiti and Turkey, but much higher than the United States [17]. Chronic heart failure, intra-cardiac thrombus, thrombo-embolic phenomena and pulmonary hypertension occurred with greater frequency in patients diagnosed with PPCM. These grave sequelae have been previously reported on in PPCM, and shown to be associated with increased mortality [4,7,16,49]. However, the seriousness of HHFP should not be under-estimated: a recent report on maternal deaths from South Africa showed that preeclampsia/eclampsia and proteinuric hypertension accounted for 83.1% of all PIH-related deaths, and that HHFP was the cause of death in 22.8% of PIH-related deaths in the same period [50].

The data from this study show that there are significant differences in the time of onset of heart failure, clinical characteristics, and outcomes of patients with HHFP compared to those with truly unexplained PPCM. Hence, these data support the proposal that a history or presence of hypertension in pregnancy should exclude the diagnosis of PPCM, as the two appear to be distinct clinical conditions [2,3,10]. However, this study has a number of limitations including small sample size, being from a single centre, differences in follow-up duration between HHFP and PPCM patients, lack of repeat echocardiography in the majority of HHFP patients at follow-up and the lack of information about repeat pregnancies and the potential for confusion between the symptoms of heart failure and the often similar symptoms of normal pregnancy. It is also important to highlight that, by its very nature, a specialist clinic in a tertiary referral center has a selection bias for the cases that develop chronic PPCM, and may miss cases that demise acutely. Finally, we acknowledge that using strict cut-offs for case definition of PPCM may have led to an underestimate of its prevalence in our setting.

Despite these limitations, this work may contribute to the clarification of the case definition of PPCM by excluding patients with any form of hypertension in this group. We show that HHFP is predominantly an antepartum disease that presents mainly with pulmonary oedema, left ventricular hypertrophy, and a reversible form of heart failure in the overwhelming majority of cases. By contrast, at our centre, unexplained PPCM is exclusively a postpartum disease that is associated with distinct clinical features of heart muscle disease (such as left bundle branch block and atrial fibrillation), chronic heart failure, and a fatal outcome in a proportion of cases. Large prospective multicenter studies of peripartum heart failure with and without hypertension are required to confirm the findings of this study.
